# Book Reviews

**Published:** 1914-10

**Authors:** 


### BOOK REVIEWS

---

Books mentioned may be inspected at and ordered through this office. So far as possible, books received in any month will be reviewed in the issue of the second month following.

---

**Polk's Medical Register and Directory of North America, 1914,** 13th edition, 1914, R. L. Polk & Co., Detroit, 2,308 pages, $10.

This directory is standard. It lists physicians by states and provinces, etc., of Canada and British America, gives a digest of the medical laws of each state and province, lists medical journals, colleges, sanatariums and hospitals, societies, etc., government services, and concludes with an alphabetic list of all physicians and a reference to pages where they are listed. Invaluable as a directory, it may also be used as the basis of interesting economic statistics. It will be seen that the physicians of the U. S. have increased by 3,768 in two years—2.922%. Assuming that the population is increasing as for the decade 1900-1910 at the rate of 21% for a decade, this represents an annual increase of about 1.924% annually, according to the formula for compound interest. It is gratifying to note that the population is increasing considerably faster than the profession. The ratios calculated for the last previous directory did not take into account the increase in population for the two years from 1910 to 1912. Not enough change has occurred to warrant the calculation of ratios for states and it should be stated that the ratios calculated for the list of 1912 was not corrected for increase in population since the census of 1910. The population of the U. S. in 1914, at the previous rate of increase (with allowance for compounding) is 99,238,076, exclusive of outlying territories. The ratio of physicians to population is, therefore, at present, 1 to 747. However, as the Polk Directory aims to list all medical graduates and includes a good many married women physicians, teachers and business men not in practice, as well as retired physicians and recent graduates not yet in private practice, it is probable that the true economic ratio is somewhere near 1 to 800.

| State | Population - 1910 | 1912 Number of Physicians | 1912 Ratio (Average Medical Clintele) | 1914 Number of Physicians |
|---|---|---|---|---|
| Alabama ......... | 2,138,093 | 2,303 | 928 | 2,505 |
| Arizona .......... | 204,352 | 260 | 786 | 278 |
| Arkansas ......... | 1,574,449 | 1,952 | 801 | 2,379 |
| California ........ | 2,377,549 | 4,425 | 537 | 4,170 |
| Colorado ......... | 799,024 | 1,593 | 501 | 1,653 |
| Connecticut ...... | 1,114,756 | 1,457 | 765 | 1,565 |
| Delaware ......... | 202,322 | 256 | 790 | 265 |
| Dist. of Columbia.. | 331,069 | 1,027 | 322 | 1,035 |
| Florida ........... | 752,619 | 996 | 756 | 1,086 |
| Georgia .......... | 2,609,121 | 2,669 | 977 | 2,848 |
| Idaho ............ | 325,594 | 400 | 814 | 436 |
| Illinois ........... | 5,638,591 | 9,679 | 583 | 10,378 |
| Indiana .......... | 2,700,876 | 4,913 | 559 | 5,095 |
| Iowa ............. | 2,224,771 | 3,604 | 617 | 3,731 |
| Kansas ........... | 1,690,949 | 2,798 | 604 | 2,802 |
| Kentucky ........ | 2,289,905 | 3,309 | 692 | 3,455 |
| Louisiana ......... | 1,656,385 | 1,860 | 890 | 2,057 |
| Maine ............ | 742,371 | 1,116 | 665 | 1,087 |
| Maryland ......... | 1,294,450 | 2,078 | 623 | 2,214 |
| Massachusetts ..... | 3,366,416 | 5,285 | 637 | 5,346 |
| Michigan ......... | 2,810,173 | 4,102 | 685 | 4,077 |
| Minnesota ........ | 2,075,708 | 2,094 | 991 | 2,377 |
| Mississippi ....... | 1,797,114 | 1,646 | 1,092 | 1,692 |
| Missouri .......... | 3,293,335 | 6,136 | 536 | 6,224 |
| Montana .......... | 376,053 | 494 | 761 | 579 |

| | | | | |
|---|---|---|---|---|
| Nebraska ........ | 1,192,214 | 1,697 | 702 | 1,875 |
| Nevada .......... | 81,875 | 130 | 629 | 153 |
| New Hampshire ... | 430,572 | 685 | 628 | 716 |
| New Jersey ....... | 2,537,167 | 2,572 | 986 | 2,715 |
| New Mexico ...... | 327,396 | 408 | 802 | 423 |
| New York ........ | 9,113,614 | 13,977 | 652 | 14,265 |
| North Carolina .... | 2,206,287 | 1,931 | 1,205 | 1,948 |
| North Dakota ..... | 577,056 | 579 | 996 | 577 |
| Ohio ............. | 4,767,121 | 7,398 | 659 | 7,204 |
| Oklahoma ........ | 1,657,155 | 2,168 | 764 | 2,356 |
| Oregon ........... | 672,765 | 937 | 718 | 985 |
| Pennsylvania ..... | 7,665,111 | 10,648 | 719 | 10,619 |
| Rhode Island ..... | 542,610 | 726 | 747 | 715 |
| South Carolina ... | 1,515,400 | 1,258 | 1,204 | 1,090 |
| South Dakota ..... | 583,888 | 678 | 866 | 670 |
| Tennessee ........ | 2,184,789 | 2,790 | 783 | 2,840 |
| Texas ........... | 3,896,542 | 5,126 | 760 | 5,171 |
| Utah ............. | 373,351 | 422 | 884 | 402 |
| Vermont ......... | 355,956 | 671 | 630 | 660 |
| Virginia .......... | 2,061,612 | 2,173 | 948 | 2,160 |
| Washington ...... | 1,141,990 | 1,533 | 745 | 1,480 |
| West Virginia ..... | 1,221,119 | 1,627 | 768 | 1,585 |
| Wisconsin ........ | 2,333,860 | 2,352 | 992 | 2,644 |
| Wyoming ........ | 145,965 | 168 | 868 | 173 |
| Totals ........ | 91,972,267 | 129,002 | 713 | 132,770 |

Continental U. S. (Exclusive of Alaska).

---

**Anoci-Association.** By George W. Crile, M. D., Professor of Surgery, School of Medicine, Western Reserve University, Cleveland; and William E. Lower, M. D., Associate Professor of Genito-Urinary Surgery, School of Medicine, Western Reserve University, Cleveland. Octavo of 259 pages, with original illustrations. Philadelphia and London: W. B. Saunders Company, 1914. Cloth, $3.00 net.

This book should be read by every medical man, surgeon or otherwise. Non-operators should know the principles in accordance with which surgical mortality and morbidity may be reduced and the average results actually to be expected. Moreover, the author is more than an operator. His surgery includes physiology, pathology, experimental research and clinical medicine. Thus, the principles and technic which he has elaborated apply to general medicine as well as to operations. Crile's work is so well known, although to too many only in a vague way, that we consider it unwise to discuss the work in such a way as to duplicate general impressions. He has contributed both to medical theory and to medical practice the most important work of the present century. This statement is made advisedly, with due regard to the internal secretions, immunization and some few other advances, both because these overlap the dividing line between centuries and because they have not been brought to the stage of relative completion—nothing is ever completed in medicine—and practical utility which Anoci-Association has already reached. Rather than attempt to make excerpts from the book, we urge that each physician should familiarize himself with the details of this subject, as presented by the originator himself. It is customary to temper words of high praise with adverse criticism. In this instance, we can do so only to the extent of expressing regret that the title of the book might not be altered in conformity with linguistic principles, before such a term as Anoci-association becomes firmly fixed in the medical vocabulary. But Dr. Achilles Rose has expressed the opinion that the editor is the only journalistic Casabianca left on the sinking ship of correct medical nomenclature.

---

**A Text Book of Military Hygiene and Sanitation.** By Frank R. Keefer, M. D., Lieutenant-Colonel, Medical Corps, United States Army; Professor of Military Hygiene, United States Military Academy, West Point. 12mo of 305 pages, illustrated. Philadelphia and London: W. B. Saunders Company, 1914. Cloth, $1.50 net.

This work appears at an opportune time in the world's history. Even for American physicians, especially the large number connected with the regular government services and the national guard, a state of preparedness for war is a necessity. The stress laid upon prophylaxis and upon the importance of disease rather than traumatism in war, deserves particular commendation. (See page 19). Physical training, food, clothing, etc., are fully discussed.

---

**Serology of Nervous and Mental Diseases.** By D. M. Kaplan, M. D., Director of Clinical and Research Laboratories of the Neurological Institute, New York City. Octavo of 346 pages, illustrated. Philadelphia and London: W. B. Saunders Company, 1914. Cloth, $3.50 net.

That so large a work could have been written on this phase of serology will doubtless arouse surprise in the minds of many. Yet the only material that can in any sense be considered as padding, is the bibliography of 70 pages and the presentation of such evidence and opportunity for further study needs no apology. While the Wasserman test, administration of salvarsan, etc., are awarded due space, syphilis is by no means the major topic of the book. Rhachicentesis, cytology of the spinal fluid, the relation of psychoses and organic diseases to toxic matters, the reactions of various diseases not fully understood, the amino-nitrogen content of sera, are among the interesting subjects discussed. So thorough a monograph is a valuable addition to medical literature.

---

**A Treatise on Clinical Medicine.** By William Hanna Thomson, M. D., LL. D., formerly Professor of Practice of Medicine and of Diseases of the Nervous System in the New York Academy of Medicine, etc. Octavo volume of 667 pages. Philadelphia and London: W. B. Saunders Company, 1914. Cloth, $5.00. Half Morocco, $6.50.

This work is essentially a practice of medicine yet presenting various original methods of discussion which set it apart from the ordinary run of such works and which must be followed by actual reading to be appreciated. In his very brief preface, the author states that the condition of the living patient should demand exclusive attention rather than pathologic states learned in the laboratory or at necropsy but he is laudably inconsistent in giving concise and definite and evidently personally familiar information as to pathology. Various general conditions and symptoms, such as pain and emaciation are discussed in the introduction, where also he discusses in a general way non-medicinal, medicinal and vaccine and serum therapy. The initial chapter on the mechanism of surface chill or catching cold is especially interesting and novel. It is stated that, ''outside of hot, moist climates, the most common cause of disease and death is from catching cold.'' This is typic of the statements made throughout the book. Here is another, alluding to a case of severe diphtheria: ''I, therefore, directed that we should wash out the throat as we would clean a dirty side-walk with a hose. A fountain-bag containing 2 gallons was suspended 6 feet over the head of the child and a steady stream of hot water with a teaspoonful of chlorate of potash and five drops of oil of peppermint to the gallon was poured into the throat . . . this douching every two hours brought away immense quantities of the membrane . . . and the child perfectly recovered.'' One may not always agree with opinions but, when definitely and succinctly expressed, with the backing of evidence and when they frequently suggest new and sometimes heterodox ideas, they are of immense value. It is a safe conclusion that this book was not written to order, to crowd into an overfull market, according to the method of spreading out a number of standard text books and dictating, with change of language, excerpts, to a stenographer.

**Collected Papers by the Staff of St. Mary's Hospital (Mayo Clinic) for 1913.** Octavo of 819 pages, 335 illustrations. Philadelphia and London: W. B. Saunders Company, 1914. Cloth, $5.50 net.

This volume maintains the high standard of its predecessors. The number and variety of the subjects treated renders review difficult. About a third of the volume is devoted to the alimentary tract but the uro-genital organs, ductless glands, head, trunk and extremities, and more general discussions of technic, after-care, notes of foreign clinics, etc., are also important parts of the contents. What renders the work especially valuable is that it is almost entirely clinical, though in no narrow sense. Perhaps a more correct statement would be that the work is based on carefully studied evidence and on things actually done or noted. The correlation of clinical study in the limited sense, of laboratory investigation and of surgery which has marked previous publications is equally conspicuous in this volume.

---

**The Practice of Surgery.** By James G. Mumford, M. D., Lecturer on Surgery in Harvard University. Second Edition, Thoroughly Revised. Octavo volume of 1,032 pages with 683 illustrations. Philadelphia and London: W. B. Saunders Company, 1914. Cloth, $7.00. Half Morocco, $8.50.

It gives us special pleasure to review so excellent a work by a resident of our own region, Dr. Mumford being surgeon to the Clifton Springs Sanitarium. The book is a complete, systematic, copiously illustrated treatise on surgery, of the highest order.

---

**Les Caracteres Medicaux Dans L'Ecriture Chinoise.** Dr. Lucien Graux, director of Gazette Medicale de Paris and La Plus Grande France, published by A. Maloine, Paris, 276 pages, paper cover, 270 cuts from drawings by Ma-Si-Tsan. 4 francs.

The author has performed a task of great historic value in compiling the Chinese graphic representations of terms used in medicine. Chinese views upon medical subjects are also given and are very curious.

---

**Annual Report of the Department of Health of Buffalo, 1913.**

This report reflects great credit upon Commissioner Francis E. Fronczak and his staff. The department employs a total force of 163 at an expense of $121,251 in salaries alone, the total cost of the department being nearly $150,000 or about 30 cents per capita. The comparatively small amount spent beyond salaries indicates great economy although it is only fair to state that this department is, in a sense, an executive agency for certain others, as in regard to the inspection and control of the sick poor, the medical examination of school children, etc. At a conservative estimate, the department of health returns the full amount invested in it, each month. To a large degree, this return to the people is at the expense of physicians, druggists and undertakers, which not even members of these professions will regret. The details of the work of the department, especially in school inspection, laboratory diagnosis, etc., deserve the attention of all physicians who are referred to the report itself.

---

**The Question of Alcohol.** By Edward Huntington Williams, M. D., published by the Goodhue Co., 120 West 32nd Street, N. Y., 121 pages, 75 cents.

This book contains essays on the following topics: The Drug Habit Menace, Temperance Instruction in Public Schools and Its Results, Liquor Legislation and Insanity, The Liquor Question in Medicine, What Shall We do About It? The author shows how the liquor consumption has increased in spite of education and legislation, deprecates the false statements of coercive methods thus far employed and attempts to solve the problems by conservative suggestions. It is a sensible discussion of a trite subject but which cannot be dismissed until some radical reform has been accomplished.

---

**Diseases of Bones and Joints.** By Leonard W. Ely, M. D., Associate Professor of Surgery, Leland Stanford Junior University, San Francisco, Cal. Sextodecimo, 220 pages, 94 illustrations. Surgery Publishing Co., New York. Cloth, $2.00.

This work, while intended for the general practitioner, is of a highly scientific nature, the illustrations being mainly photo-micrograph reproductions and the principles of pathology and therapeutics involved being presented rather than a confusing mass of "practical" detail. Reference is facilitated by marginal headings in red.

---

**Guiding Principles in Surgical Practice.** By Frederick-Emil Neef, B. S., M. L., M. D., Adjunct Professor of Gynecology, Fordham University School of Medicine, New York City. Sextodecimo, 180 pages. Surgery Publishing Co., New York City. Cloth, $1.50.

Perhaps the best understanding of what this book covers can be afforded by citing the marginal references, which, as in the preceding, are in red. Logical asepsis, extrinsic and intrinsic infection, pre-operative fever, general and local preparation, cleansing and disinfection, cleansing instead of disinfection, preparation of the mucous membrane, nurses' outline of preparations, procedure in the operating room, disposal of soiled wash, arrangement of the supply table, repair of smooth muscle, heart muscle, nerve tissue, etc., microscopic changes during catgut absorption. These are only a few from the beginning of the book. While the work makes no pretense at being a manual of surgery, the problems liable to arise at any stage of the conduct of a case are similarly treated and in logical order.

---

**A Treatise on Diseases of the Nose, Throat and Ear.** By William Lincoln Ballenger, M. D., Professor of Laryngology, Rhinology and Otology in the College of Physicians and Surgeons, Chicago. New (4th) edition, thoroughly revised. Octavo, 1080 pages, with 536 engravings, mostly original, and 33 plates. Cloth, $5.50 net. Lea & Febiger, Philadelphia and New York, 1914.

This is an elaborate systematic treatise covering the generalized specialty of the three organs as it has now developed from clinical experience. Anatomy is briefly discussed in a preliminary chapter, minute details and histology being considered along with pathology, as problems arise. The work is well balanced between operative and local or even general therapeutics. The chapter on office equipment is very practical and avoids the extremes of encouraging the beginner to attempt specialization with an inadequate outfit and of advertising the author's ability of indulging in every last luxury. The illustrations are so copious that we even have a picture showing how to moisten a pledget of cotton by inverting a vial over it.

---

**U. S. Brewers' Association: Year Book,** with proceedings of 43d annual convention at Atlantic City, October, 1913. 311 pages, not copyrighted.

This book is largely of a propagandist nature. Its obvious intention is to further the consumption of malt liquors and, whatever opinion one may hold on this matter, it is necessarily involved in the broader one of whether individual freedom is to be controlled by majority vote. 63,283,123 barrels of fermented liquors were produced in the United States in 1911; 62,176,694 in 1912. In regard to the claim that brewers have multiplied drinking places abnormally, it is interesting to note that the internal revenue statistics of persons paying taxes as retail liquor dealers, exclusive of prohibition states, have decreased from 219,800 in 1908 to 207,764 in 1912. The statement that the United States ranks first as a beer drinking nation seems to be refuted by the latest available general statistics (1909) which show it to be tied with Sweden for sixth place, on a per capita average of 1.14 gallons a year, Denmark leading with an average of 2.16 gallons.

---

**Diphtheria: Its Prevention and Control.** By J. W. Schereschewsky, U. S. Public Health Service, Supplement No. 14 to the Public Health Reports, April 17, 1914. Price 5 cents.

This is a comprehensive up-to-date review of this subject and should be in the hands of every sanitary inspector, nurse and teacher to all of whom as well as to the physician it will give a clear understanding of the modern way of controlling this disease.—C. G. L-W.

---

**The Therapeutic Value of the Potato.** By Heaton C. Howard, L. R. C. P., M. R. C. S., published by Paul B. Hoeber, 69 East 59th Street, New York City, pamphlet, 31 pages, illustrated, 50 cents.

As the potato is a solanum, it is obviously of some narcotic value, so far as its green parts are concerned. Its salts are also of value as antiscorbutics. It was anciently used as a poultice in South America but the allusions to other solanaceae by writers in Europe prior to the discovery of America must be considered merely as contributory evidence. The author discusses the value of potato extracts, free from starch and nitrogenous matter, in synovitis, gout, lumbago, rheumatism and bruises. Liquid extract, ointment, plaster, liniment and ampulla for hypodermatic use, are described.

---

**Blood Pressure: Its Clinical Applications.** By George W. Norris, A. B., M. D., Assistant Professor of Medicine in the University of Pennsylvania; Visiting Physician to the Pennsylvania Hospital; Assistant Visiting Physician to the University Hospital; Fellow of the College of Physicians of Philadelphia. Octavo, 372 pages, with 98 engravings and 1 colored plate. Cloth, $3.00 net. Lea & Febiger, Publishers, Philadelphia and New York, 1914.

About half of the book is devoted to the physics and technic of sphygmomanometry and allied topics. It will surprise many readers to note the large number of instruments available. The author considers the mercury manameter, of portable type, with reservoir, as the best for general purposes. Instruments for graphic registration are described at length. The discussion of venous and capillary pressure, rate of blood flow, etc., is interesting even for those whose use of instruments will be limited to routine observation of arterial pressure. The physiologic and pathologic states influencing blood pressure are considered both from the diagnostic side from that of prophylaxis, therapeutics, prognosis, etc., the last implying a good though brief discussion of blood pressure in life insurance examinations.

---

**Dietetics: Or Food in Health and Disease.** By William Tibbles, LL. D., M. D., L. R. C. P., M. R. C. S., L. S. A. Medical Officer of Health, Fellow of the Royal Institute of Public Health, etc. Octavo, 627 pages. Cloth, $4.00 net. Lea & Febiger, Publishers, Philadelphia and New York, 1914.

Some books on dietetics are analogous to works on **materia medica**, listing different food stuffs either alphabetically or according to system of physiologic value and giving lengthy descriptions of these food stuffs from the botanic, historic and culinary standpoints. Others attack the general subject mainly from the standpoint of therapeutics, classifying by diseased or peculiar physiologic conditions such as infancy, lactation, old age, etc. Still others are essentially works on organic chemistry. The first part of this book is mainly devoted to the chemistry of dietetics and of physiologic processes as related to chemistry while the latter and major part is mainly therapeutic in nature. This view-point seems to us most appropriate since it gives the physician a clear insight into chemic principles of diet and then enters into details of dietetic practice, the botanic or zoologic classification of food stuffs being of comparatively little practical value and a description of potatoes, carrots, oxen, oysters, etc., in detail, and of their methods of cooking and uses, being somewhat superfluous. The author discusses the inorganic, the non-proteid but nitrogenous, the acid and other constituents of foods aside from proteid, fat and carbohydrate in a very satisfactiry way. He takes a conservative attitude regarding Chittenden's low proteid ration, distinguishing clearly between what a man may live on and what affords him the best nutrition. In his calculations of calories, etc., and especially with regard to economic dietetics, he is naturally somwhat handicapped by the non-decimal systems of Great Britain. In the table of composition of foods, he appends a column of food values per kilogram, obtained by multiplying the net, digestible number of grams of proteid, fat and carbohydrate, by 5, 3, and 1, respectively. This, or any analogous method seems to us quite arbitrary. A simple statement of calories per kilogram or gram, is a better basis of comparison. Tibbles states the calories per ounce, affording a very convenient table for direct estimation of food values. Just why the 5 to 3 to 1 ratio is adopted, is not quite clear. To say that a food stuff containing—to facilitate a simple comparison—10% of proteid is 5 times as valuable as one containing 10% of carbohydrate is, of course, quite incorrect if the immediate need is of energy, and an understatement of the fact if tissue repair is urgent. Comparing fat with carbohydrate, the former has about 2 1-3 times as much caloric value but, when we compare the average digestibility and absorbability of fats and carbohydrates, the ratio is only about 2 to 1. Considering the tendency to acid intoxication by fats, and the very limited degree to which they are assimilable, and the probability that the body may dispense with them altogether, the ultimate food value of fats, calorie for calorie, is considerably less than of carbohydrates. Now, while the caloric value of foods may be mathematically computed and compared, the ultimate food value is an entirely different conception, scarcely reducible to figures. Whether beef or sugar or butter is more valuable, is about like a similar estimate of the value of shoes, a shirt and a pair of trousers, depending upon present needs. This is, of course, a very minor point of criticism, but it indicates a very major excellence of the book. In reading this book, one gets definite statements which stimulate interest. It takes for granted matters of common knowledge and strikes at the root of dietetic principles. In the dietetic management of nutritional states and diseases, very definite information is given and various authorities are quoted in a judicial spirit.

---

**Collected Papers From the Research Laboratory of Parke, Davis & Co.**, Reprints, Vol. 2, 1914.

This volume contains 22 articles, reprinted from various scientific journals. 5 papers cover the work of Walter E. King, Robert H. Wilson, F. W. Baeslack and George L. Hoffmann, on hog cholera. H. C. Hamilton, A. W. Lescohier and R. A. Perkins claim the practical identity of American with Indian hemp—a matter of considerable importance in the present crisis when we must develop our own sources of drugs. Baeslack and H. R. Varney, make a comparative study of Wassermann antigens. Charles T. McClintock and Willard H. Hutchings describe the effects of chloretone, carbolic acid, magnesium sulphate, etc., in tetanus inoculated into sheep. They conclude that amputation at the beginning of symptoms does no good and that antetanic has a definite though usually insufficient curative effect. They consider the exhaustion from muscular contractions an important item and believe this can be controlled by chloretone. T. B. Aldrich contributes a study of the iodine content of the small, medium and large thyroids of sheep, cattle and hogs. Various other interesting scientific articles are contained in this volume.

